# Inhibins Tune the Thymocyte Selection Process by Regulating Thymic Stromal Cell Differentiation

**DOI:** 10.1155/2015/837859

**Published:** 2015-04-20

**Authors:** Ebzadrel Carbajal-Franco, Marisol de la Fuente-Granada, Germán R. Alemán-Muench, Eduardo A. García-Zepeda, Gloria Soldevila

**Affiliations:** Departamento de Inmunología, Instituto de Investigaciones Biomédicas, Universidad Nacional Autónoma de México, 04510 México, DF, Mexico

## Abstract

Inhibins and Activins are members of the TGF-*β* superfamily that regulate the differentiation of several cell types. These ligands were initially identified as hormones that regulate the hypothalamus-pituitary-gonadal axis; however, increasing evidence has demonstrated that they are key regulators in the immune system. We have previously demonstrated that Inhibins are the main Activin ligands expressed in the murine thymus and that they regulate thymocyte differentiation, promoting the DN3-DN4 transition and the selection of SP thymocytes. As Inhibins are mainly produced by thymic stromal cells, which also express Activin receptors and Smad proteins, we hypothesized that Inhibins might play a role in stromal cell differentiation and function. Here, we demonstrate that, in the absence of Inhibins, thymic conventional dendritic cells display reduced levels of MHC Class II (MHCII) and CD86. In addition, the ratio between cTECs and mTECs was affected, indicating that mTEC differentiation was favoured and cTEC diminished in the absence of Inhibins. These changes appeared to impact thymocyte selection leading to a decreased selection of CD4SP thymocytes and increased generation of natural regulatory T cells. These findings demonstrate that Inhibins tune the T cell selection process by regulating both thymocyte and stromal cell differentiation.

## 1. Introduction

Inhibins are members of the TGF-*β* superfamily that regulate different cellular functions including proliferation, apoptosis, and differentiation of several cell types and play a role in the immune system (reviewed in [1]).

Activins and Inhibins were first described as hormones that regulate FSH release by the pituitary, either activating or inhibiting its release, respectively. One of the evidences that supported the role of Inhibins as antagonists of Activins was the fact that Inhibin *α*-subunit deficient mice (Inh*α* ^−/−^) develop gonadal tumors and cachexia with severe weight loss and liver necrosis [2, 3]. Some of the observed symptoms in Inh*α* ^−/−^ appeared to correlate with the presence of high levels of serum Activin after 6 weeks of age [3]. However, growing evidence indicates that Inhibins do not always antagonize Activin-mediated functions, arguing in favor of a putative independent signaling pathway for Inhibins (reviewed in [4]).

Recent data has shown that Activins and Inhibins regulate the differentiation of several immune cell types including macrophages, dendritic cells (DCs), and T cells (reviewed in [5, 6]). For example, Activin/Inhibin signaling has been shown to regulate DC functions in steady-state and inflammatory conditions through several mechanisms (reviewed in [5]). Interestingly, both Activin and Inhibin were shown to impair DC maturation* in vitro*, further arguing against the role of Inhibins as mere antagonists of Activin functions in immune cells [7].

T cell development is a highly regulated process that requires the close interaction between immature thymocytes and stromal thymic cells. Both cell-cell contact interactions and the presence of soluble mediators (cytokines, chemokines, and growth factors), mainly produced by stromal cells, regulate thymocyte differentiation and migration [8]. In the mouse, thymocyte development is initiated during fetal life with the arrival of lymphoid progenitors from the fetal liver that seed the thymic epithelial rudiment around day 11 of gestation (reviewed in [9]). After birth, bone marrow (BM) derived progenitors reach the thymus through blood vessels located at the corticomedullary junction [10]. The most immature thymocytes (DN or CD4^−^CD8^−^ double negative) migrate outwards through the thymic cortex until they reach the subcapsular region (reviewed in [11, 12]). Pre-TCR signaling allows the differentiation of DN to the double positive stage (DP, CD4^+^CD8^+^), where expression of a functional TCR allows thymocytes to interact with self-peptide-Major Histocompatibility Complex (MHC) complexes expressed on cortical thymic epithelial cells (cTECs) and rescue them from apoptosis by the process of positive selection, while those thymocytes whose TCRs are unable to recognize self-peptide-MHC ligands die by neglect. Positively selected thymocytes migrate towards the medulla while they downregulate the CD4 or CD8 coreceptor, depending on the TCR specificity becoming CD4SP or CD8SP (SP, single positive) [13].

The thymic stroma consists of a heterogeneous population of epithelial and BM derived cells that provide the specific microenvironment required to support T cell differentiation in the thymus [12]. Among epithelial cells, cTECs and medullary epithelial cells (mTECs) are originated from a common bipotent precursor of endoderm origin which simultaneously expresses the cTEC marker CD205 and the mTEC regulator Receptor Activator of NF-*κ*B (RANK) ([14] and reviewed in [15]). Among BM derived cells, DCs and macrophages are abundant in the thymic stroma. Three types of DCs, migratory (CD8-*α* ^−^ SIRP-*α* ^+^) and resident (CD8-*α* ^+^ SIRP-*α* ^−^) conventional DCs (cDCs) and plasmacytoid DCs (pDCs) have been described in the thymus (reviewed in [16, 17]). Non-migratory cDCs are generated in the thymus and their development parallels the kinetics of thymocyte development following the arrival of BM progenitors, while migratory DCs include pDC and the CD8*α* ^−^ SIRP-*α* ^+^ cDCs, which arrive to the thymus as preformed DCs from the bloodstream [18].

cTECs mediate positive selection of DP immature thymocytes in the cortex while mTECs and DCs are involved in negative selection of potentially autoreactive T cells, ensuring the generation of a T cell repertoire highly diverse but devoid of self-reactivity. In addition to a special CD4^+^ subpopulation, the naturally occurring regulatory T cells (nTregs) are also generated in the thymus under conditions of high avidity, mostly selected by mTECs and DCs present in the medulla [19]. On the other hand, pDCs also play a role in negative selection [20] and in the generation of nTregs [21]. Finally, macrophages are considered scavenger cells, responsible for the elimination of apoptotic cells that fail to undergo positive selection in the cortex, as well as those cells that die in the medulla as a result of the negative selection process [22, 23].

TGF-*β* superfamily members regulate specific checkpoints of thymocyte differentiation (reviewed in [1]). In this context, we previously reported that Inhibins are the major Activin ligands expressed in the thymus [24]. Moreover, both Inhibins and Activins promote DN to DP thymocyte differentiation during* in vitro* T cell development of murine fetal thymocytes. In contrast, Activins, but not Inhibins, promoted the transition from DN to intermediate single positive (ISP, CD8^+^) stage, indicating that these ligands may exert different actions depending on the differentiation state of the cell [25]. In addition, both ligands (Activins and Inhibins) and Activin receptors (ALK4, ActRIIA, and ActRIIB) are expressed by stromal cells in the cortical and medullary regions of the murine thymus [24, 26]. As these ligands can act both in a paracrine and autocrine manner in different cell types [5], here we investigated whether Inhibins may be involved in stromal cells differentiation and function.

## 2. Materials and Methods

### 2.1. Mice

Inhibin *α* heterozygous mice (Inh*α* ^+/−^) were previously described [2]. Mice were bred and maintained at the Instituto de Investigaciones Biomédicas (IIB, UNAM, Mexico) animal breeding facility in SPF conditions, according to ethics guidelines. The study was approved by the “Comité para el Cuidado y Uso de Animales de Laboratorio (CICUAL)” of the IIB. Heterozygous Inh*α* ^+/−^ mice were intercrossed to generate homozygous wild type (Inh*α* ^+/+^) or knockout mice (Inh*α* ^−/−^). For all experiments, 2-week-old female mice were used.

### 2.2. Immunohistochemistry (IHC)

*MHCII Detection*. Paraffin-embedded thymi from 2-week-old female mice were fixed in 4% paraformaldehyde in PBS (pH 7.4) at 4°C for 18 h and dehydrated (using an automated Leica Tissue Processor). Then tissues were sliced (3 *μ*m) and dewaxed in xylene and gradually rehydrated with graded ethanol solutions and then washed with PBS for further rehydration. For antigen unmasking Immuno/DNA retriever with citrate (Bio SB) was used and then sections were exposed to dry heat (65°C) for 35 min. Sections were subsequently washed with PBS and incubated with Peroxidazed 1 solution (Biocare Medical, Concord, CA, USA) for 5 min to quench endogenous peroxidases. The samples were washed with PBS and incubated for 10 min at RT with a protein-blocking solution consisting of PBS containing 1% BSA. Sections were then incubated with a 1 : 100 biotinylated monoclonal rat anti-mouse I-A/I-E (BD Biosciences, San Jose, CA, USA) antibody overnight at 4°C. After three PBS washes, sections were incubated with the 4+ Streptavidin HRP label (Biocare Medical) for 20 min at RT. The DAB chromogen kit (Biocare Medical) was applied to tissue sections for 45 sec to detect MHCII positive cells and counterstained with CAT Hematoxylin (Biocare Medical). Finally, all slides were dehydrated through graded alcohols to xylene and coverslipped using Entellan (Merck, Frankfurt, Germany).

*CD11c Detection.* Thymi were fixed as before and dehydrated at 4°C in sucrose (10, 20, and 30% in PBS). Tissues were snap-frozen in OCT compound (Tissue-Tek, Sakura). 8 *μ*m thick tissue sections were rinsed with PBS and endogenous peroxidase was blocked by use of 0.28% periodic acid in deionized water for 45 sec at RT. The samples were washed with PBS and a protein-blocking solution was used. Sections were then incubated overnight with a 1 : 50 biotinylated monoclonal hamster anti-mouse CD11c (BD Biosciences). As for Class II analysis, the sections were stained with 4+ Streptavidin HRP, DAB, and counterstained with CAT Hematoxylin. Previously dehydrated with graded alcohols to xylene, all slides were coverslipped using Entellan.

*Analysis of MHCII and CD11c Expression.* Positive staining was identified by the presence of ochre color. For each molecule, micrographs of five fields were taken from each thymic section with a light Olympus CX 31 microscope coupled with an Olympus C-7070 digital camera (Olympus, Tokyo, Japan). Quantitation of the positively stained area and signal intensity (from 40x micrographs) was performed with the ImageJ 1.46r software (National Institutes of Health, USA).

### 2.3. Thymic Stromal Cell Isolation for Flow Cytometry Analysis

*cDCs and pDCs Isolation*. Thymi from 2-week-old female mice were gently separated. Each thymus was placed in a Petri dish with 3 mL of RPMI-1640 containing 0.125% Collagenase type IV (Invitrogen, Life Technologies, Carlsbad, CA,USA) and 25 U/mL of Dnase I (Roche, Basel, Switzerland). Thymus was cut into small fragments and incubated for 1 h at 37°C, 5% CO_2_ in a humidified atmosphere. Then, the thymic fragments were disaggregated using a syringe plunger and the cellular suspension was filtered through 150 *μ*m nylon mesh. Finally, the cells were washed and resuspended in cold EDTA/FACS buffer.

### 2.4. Thymic Epithelial Cell (TEC) Isolation

Enzymatic digestion of thymus was adapted from a previously described protocol [27]. Briefly, each thymic lobe was nicked, placed in a conical tube with 5 mL of RPMI-1640, and gently vortexed for several minutes to flush out as many thymocytes as possible. The supernatant was collected on ice and replaced as it became visibly cloudy. The thymic remnants were then incubated at 37°C for 15 minutes in 2 mL of 0.125% (w/v) Collagenase type IV (Invitrogen, Life Technologies) and 25 U/mL of DNase I (Roche) in RPMI-1640 with regular gentle agitation. Fragments were allowed to settle. The supernatant was collected and kept on ice, and the digestion was repeated 3 times using the remaining settled thymic fragments. Lastly, remaining thymic fragments were incubed for 15 min, or until dispersed, with 3 mL of 0.125% (w/v) Collagenase/Dispase (Roche) with 25 U/mL of DNase I (Roche) in RPMI-1640. Cells from all supernatant fractions were washed, pooled, and resuspended in cold EDTA/FACS buffer and filtered through 150 *μ*m nylon mesh. Finally, cells were counted using a hemocytometer and dead cells were excluded by trypan blue staining.

### 2.5. Flow Cytometry

For phenotypic analysis of the isolated thymic stromal cells, they were incubated with primary antibodies for 30 min at 4°C, followed by two washes with EDTA/FACS buffer. When necessary, fluorochrome-coupled secondary antibody or fluorochrome-coupled streptavidin was added, incubated for 30 min at 4°C, and washed. For analysis of cDCs the following primary antibodies and reagents were used: anti-CD11c-PE (BioLegend, San Diego, CA, USA), anti-CD86-APC (BioLegend), anti-CD80-PerCP/Cy5.5 (BioLegend), anti-IA/IE-Biotin (BD Biosciences), and Streptavidin-FITC (SIGMA, Saint Louis, MO, USA). As primary antibodies, anti-CD11c-PE, anti-IA/IE-Biotin, Streptavidin-APC-Cy7, and anti-CD45R/B220-FITC (all from BD Biosciences) were used to examine thymic pDCs. For analysis of thymic epithelial cells anti-CD45-PerCP (BD Biosciences), anti-IA/IE-Alexa Fluor 488 (BioLegend), and anti-Ly-51-Alexa Fluor 647 (BioLegend) were used.

For intracellular Activin A detection, after surface staining for subpopulation markers, cells were permeabilized using fixation/permeabilization buffer (eBioscience, San Diego, CA, USA) for 2 h. Subsequently, the Fc receptors were blocked with anti-mouse CD16/32 (BioLegend) and murine serum and then incubated with anti-human/mouse/rat Activin A (R&D Systems, Minneapolis, MN, USA) followed by goat anti-mouse IgG-Alexa Fluor 488 antibody (Invitrogen, Life Technologies).

For thymocyte subpopulations and regulatory T cells anti-CD25-FITC (BD Biosciences), anti-CD8-PerCPCy5.5 (BioLegend), anti-Foxp3-APC (eBioscience), and anti-CD4-APC-Alexa Fluor 750 (Invitrogen, Life Technologies) were used. For the intracellular staining of Foxp3, fixation/permeabilization buffer (eBioscience) was used according to the manufacturer instructions.

All samples were acquired in an Attune Acoustic Focusing Flow Cytometer (Life Technologies). Dead cells were gated out based on forward scatter (FSC) and side scatter (SSC). The data were analyzed using FlowJo 7.6 software (Tree Star Inc.).

### 2.6. Activin A ELISA

Serum samples of mice were assayed for Activin A using the Human/Mouse/Rat Activin A Quantikine ELISA Kit (R&D systems) following the instructions of the manufacturer. Briefly, biotinylated capture antibody was placed into 96-well microtiter streptavidin-coated plates and incubated at RT for 15 min. After a wash, standards and samples were added into the wells and were incubated for 3 h at RT on a horizontal orbital microplate shaker. After washing, anti Activin *β*A subunit antibody conjugated to HRP was added, followed by substrate solution. Colorimetric analysis was performed using a Modulus II Microplate Multimode Reader (Turner Biosystems Inc., Sunnyvale, CA, USA).

### 2.7. Statistical Analysis

Data were expressed as mean values ± SEM. For all the experiments, a Student's* t* test (two tailed, paired or unpaired) was used. Asterisk (∗) indicates statistically significant differences (*P* ≤ 0.05).

## 3. Results and Discussion

### 3.1. Thymic Stromal Cells of Inh*α* ^**−**/**−**^ Mice Show Reduced Levels of MHCII Molecules

**Figure 1:**
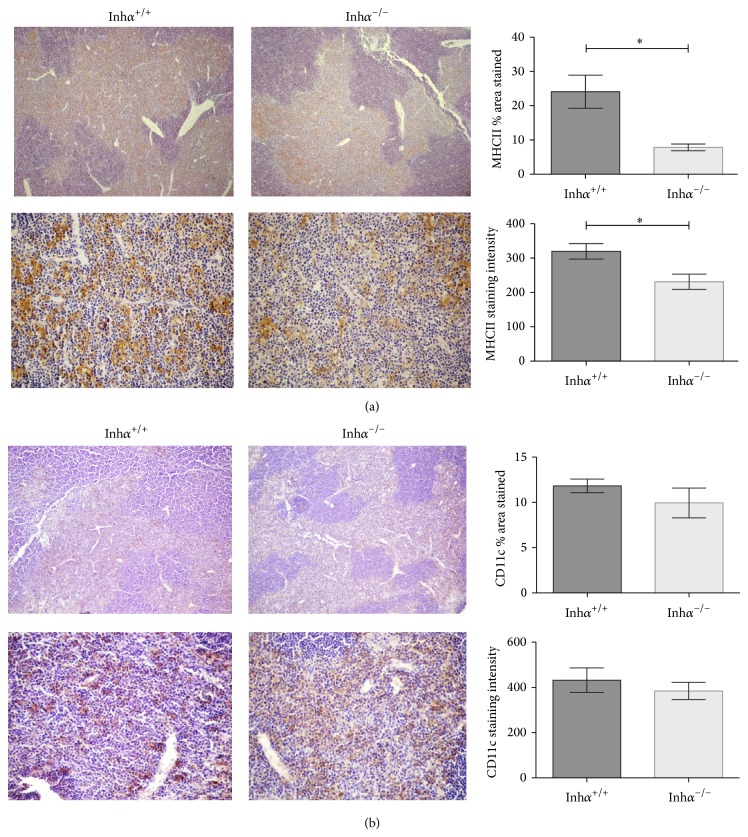
MHCII expression but not CD11c^+^ cells is reduced in thymus of Inh*α* ^−/−^ mice. Representative micrographs of thymic sections from 2-week-old Inh*α* ^+/+^ (*n* = 3) and Inh*α* ^−/−^ (*n* = 6) mice stained for MHCII (a) and CD11c (b) are shown at 5x (top panels) and 40x (bottom panels) magnification. (a) MHCII staining and summary of data expressing the percentage of area stained per field (top, *P* = 0.002) and the MHCII staining intensity (bottom, *P* = 0.04) are shown. (b) CD11c staining and summary of data expressing the percentage of area stained per field (top) and the CD11c staining intensity (bottom). Values are expressed as mean ± SEM. Statistical significance: ^∗^ *P* ≤ 0.05.

We have previously demonstrated that Inh*α* ^−/−^ mice have delayed T cell development mainly at the DN to DP transition and reduced thymocyte numbers [25]. As engagement of endogenous peptide-MHCII complexes by the TCRs expressed on developing thymocytes is crucial for their selection and survival [8], we analyzed the expression of MHCII in the thymic stroma of 2-week-old Inh*α* ^−/−^ mice or Inh*α* ^+/+^. This age was selected to minimize the possible interference of intrinsic factors present in the Inh*α* ^−/−^ mice, since it has been reported that this mouse develops gonadal sex cord-stromal tumors as early as 4 weeks of age which cause cancer related cachexia-like symptoms [3, 28]. Immunohistochemical analysis showed that thymi of Inh*α* ^−/−^ mice expressed reduced levels of MHCII molecules, which was evident in the thymic medulla (Figure 1(a)). Although cTECs express both MHCI and MHCII molecules, the levels of MHCII staining in the cortex are lower than in the medulla, possibly due to thymocyte masking of MHCII as a result of the smaller proportion of cTECs/thymocytes in the cortex [29]. Therefore, by this technique we cannot rule out the possibility that cortical stromal cells could also show alterations in their MHCII expression.

MHCII-expressing cells in the thymus include cDCs, pDCs, macrophages, epithelial cells, and B cells [30]. To determine the subpopulation responsible for the diminished expression of MHCII, we analyzed the presence of thymic DCs by IHC using the CD11c marker [31, 32]. Our results indicated that there was a slight decrease, although not significant, in CD11c^+^ cells in Inh*α* ^−/−^ thymi compared to Inh*α* ^+/+^ (Figure 1(b)), suggesting that the difference in MHCII could not merely be attributed to DCs.

### 3.2. Inh*α* ^**−**/**−**^ Thymic cDCs Display Reduced Levels of MHCII and Costimulatory Molecules

**Figure 2:**
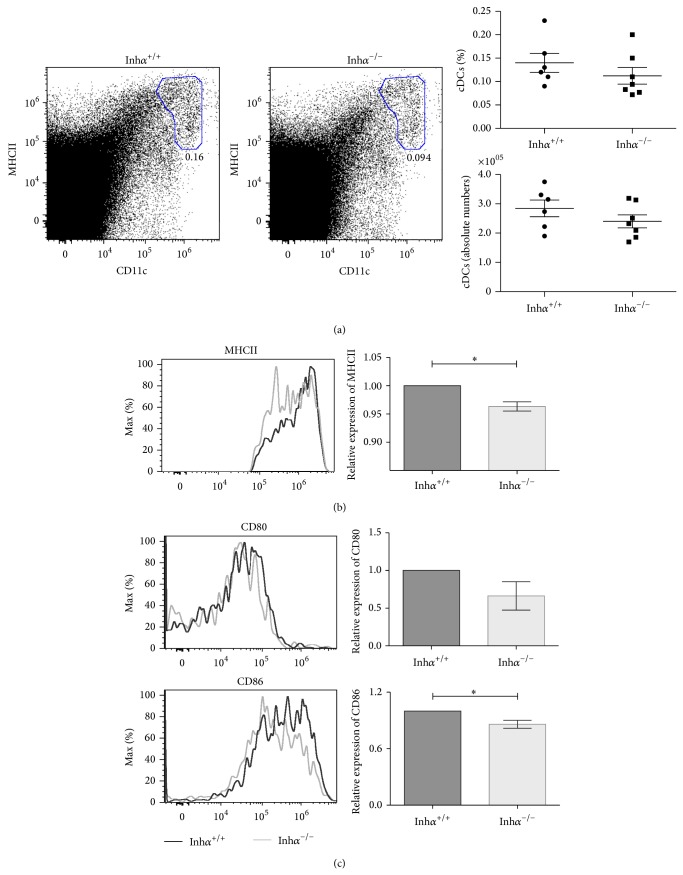
MHCII and CD86 expression in cDCs are reduced in thymus of Inh*α* ^−/−^ mice. (a) Representative flow cytometry dot plots and frequency of thymic cDCs defined as CD11c^+^ MHCII^+^ cells. For the MHCII (b), CD80 (c), and CD86 (c) expression, MFI was determined and reported as relative expression compared to Inh*α* ^+/+^ mice. A significant reduction was observed for MHCII (*P* = 0.04) and CD86 (*P* = 0.04) expression in Inh*α* ^−/−^ thymic cDCs. One representative example of a total of 13 mice is shown (Inh*α* ^+/+^, *n* = 6; Inh*α* ^−/−^, *n* = 7) from 3 independent experiments. Values are expressed as mean ± SEM. Statistical significance: ^∗^ *P* ≤ 0.05.

To further analyze hematopoietic derived thymic stromal cells, thymi were disaggregated through mechanical and enzymatic methods and analyzed by flow cytometry to identify cDCs, pDCs, and macrophages. In agreement with our previous data (Figure 1(b)), we observed a small reduction, although not significant, in the percentage and numbers of cDCs (CD11c^hi^ MHCII^hi^) in Inh*α* ^−/−^ compared with Inh*α* ^+/+^ mice (Figure 2(a)). Importantly, in this subpopulation we found a significant reduction in the mean fluorescence intensity (MFI) of MHCII when comparing Inh*α* ^−/−^ with Inh*α* ^+/+^ (Figure 2(b)), suggesting that Inhibins may also be involved in the maturation of cDCs. Next, we analyzed the expression of costimulatory molecules CD80 and CD86, upregulated during DC maturation [31] and known to mediate the negative selection as well as the generation of nTregs (reviewed in [33]). As shown in Figure 2(c), CD86, but not CD80 levels, were significantly reduced in Inh*α* ^−/−^ compared to Inh*α* ^+/+^ cDCs. The reduction of MHCII in the absence of Inhibins opposes previously reported data showing that Inhibin A is capable of preventing the upregulation of HLA-DR expression during human DC maturation* in vitro* [7]. However, it is worth noting that Activin ligands may exert different effects on immune cells under either inflammatory [34, 35] or steady state conditions [36], as the data shown here.

**Figure 3:**
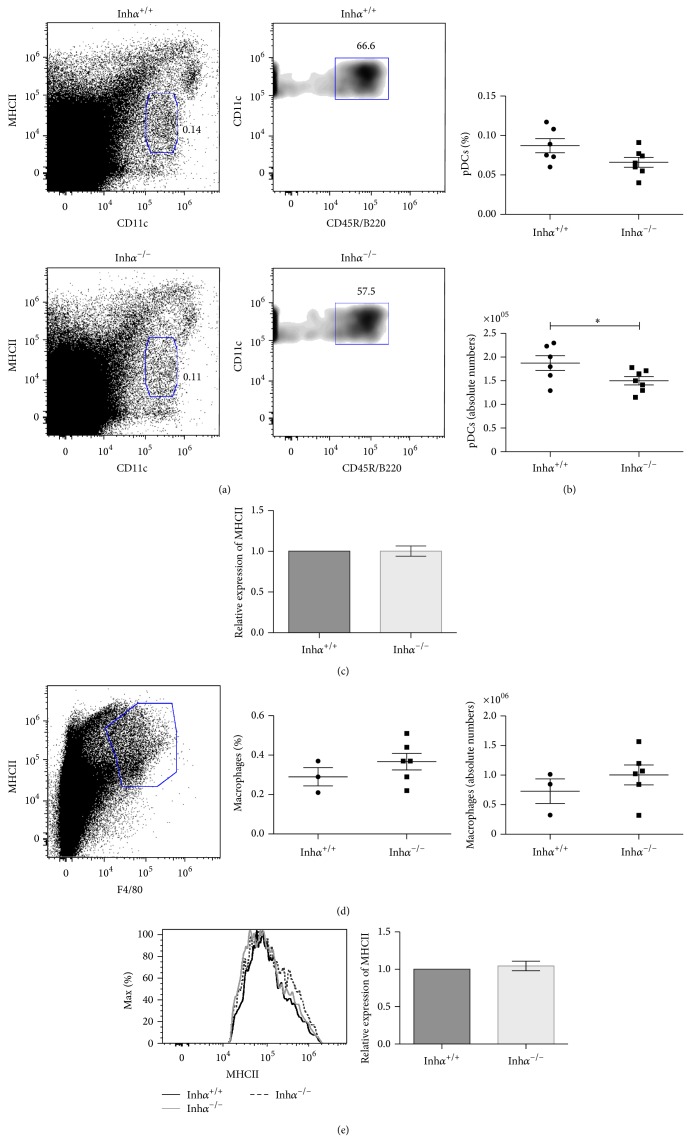
In Inh*α* ^−/−^ mice absolute pDCs numbers are diminished in thymus, while other hematopoietic derived stromal cells do not show alterations (macrophages). (a) Representative dot plots of the strategy used to analyze pDCs; from total thymus an initial gate of CD11c^med^ MHCII^lo^ cells was used (a, left) followed by a region in CD45R/B220^+^ cells (a, right). (b) Graphs represent the percentages and total numbers of thymic pDCs identified as CD11c^med^ MHCII^lo^ CD45R/B220^+^ cells. (c) Expression of MHCII on pDCs. MFI was determined and relative expression calculated as in Figure 2. For pDCs, Inh*α* ^+/+^, *n* = 6 and Inh*α* ^−/−^, *n* = 7 were analyzed. (d) Analysis of macrophages was performed using a CD45^+^ gate, followed by MHCII^+^ F4/80^+^ detection. Percentages and absolute numbers are shown. (e) A representative histogram showing the MHCII expression in macrophages of Inh*α* ^+/+^ and Inh*α* ^−/−^ mice (left panel); for each histogram two Inh*α* ^−/−^ (gray and dashed lines) and one Inh*α* ^+/+^ mice (black line) were represented and the summary of data showing the relative expression of MHCII in macrophages (right panel) is shown. For macrophages, Inh*α* ^+/+^, *n* = 3 and Inh*α* ^−/−^, *n* = 6 were used. Statistical significance: ^∗^ *P* ≤ 0.05.

Next, we analyzed thymic pDCs which comprise the ~0.04–0.12% of total thymic cells [37, 38] and 30% of the total thymic DCs (reviewed in [39]). Similarly to the effect observed in cDCs, the percentage of pDCs, defined as CD11c^med^ MHCII^lo^ CD45R/B220^+^ cells (Figure 3(a)) [31, 40], is slightly lower in Inh*α* ^−/−^ compared with Inh*α* ^+/+^ mice (0.07% ± 0.006% versus 0.09% ± 0.009%, *P* = 0.07) (Figure 3(b)). Interestingly, there was a significant decrease in total numbers of thymic pDCs in Inh*α* ^−/−^ mice compared to Inh*α* ^+/+^ (149,890 cells ± 8,697 cells versus 187,202 cells ± 15,684 cells, *P* = 0.05) (Figure 3(b)). However, in contrast to that observed in cDCs, the levels of MHCII in Inh*α* ^−/−^ pDCs were similar to those in Inh*α* ^+/+^ thymi (Figure 3(c)). These differences may be explained by the fact that expression of the MHCII transactivator (CIITA), the master regulator of MHCII, is controlled by different promoter regions in different cell types. Specifically, CIITA expression on pDCs relies on the B cell promoter pIII whereas all other DCs depend on pI [41].

The differences observed in the numbers and proportions of thymic DC subpopulations in Inh*α* ^−/−^ mice may involve alterations in DC differentiation and/or homing. It has been described that homing of distinct DC thymic subsets depends on specific receptors. For CD8-*α* ^−^ SIRP-*α* ^+^ migratory cDCs, expression of CCR2 (and its ligand CCL8) is required for their intrathymic localization. On the other hand, CCR7 and CCR9 were shown to be crucial for homing of T/DC common BM progenitors, that give rise to CD8-*α* ^+^ SIRP-*α* ^−^ thymic resident cDCs, while their accumulation in the medulla requires the XCR1 and its ligand XCL1 produced by mTECs MHCII^hi^ [18].

Our data showed a slight reduction in the percentage and numbers of cDCs. However, given that a subset of DCs arrive from the periphery, while others differentiate* in situ*, we cannot discriminate whether the effects observed in Inh*α* ^−/−^ mice are the result of either impaired homing of preformed DCs or altered* in situ* differentiation of BM progenitors. Additionally, the significant reduction of total numbers of thymic pDCs may also indicate an impaired homing to the thymus from the blood, which has been shown to depend on CCR9 [20]. In this context, although there is no evidence on the role of Activins/Inhibins on CCR9-mediated migration, TGF-*β*, another member of the same superfamily, was shown to upregulate CCR9 on murine T cells [42]. Therefore we cannot rule out the possibility that CCR9 expression on pDCs could also be regulated by Activin ligands.

Regarding the migration of cDCs, Activin A was shown to regulate CCR2 and CCL2 expression in human macrophages [43]. Activin A induces DC migration through the polarized release of CXCL12 and CXCL14 [44]. Moreover, Activin was shown to upregulate CXCR4 in CD40L-stimulated Langerhans cells [45]. Conversely, Activin produced by monocyte-derived DCs activated by TLR and CD40L signaling was shown to negatively regulate DC migration [46]. Therefore, Activin/Inhibin ligands may act either promoting or inhibiting cell migration depending on the cytokine milieu and the specific DC subset.

Thymic macrophages defined as MHCII^+^ F4/80^+^ cells exhibited a different pattern. Although not statistically significant, the percentage and cell numbers of macrophages were slightly higher in Inh*α* ^−/−^ compared to Inh*α* ^+/+^ mice (Figure 3(d)). The levels of MHCII in Inh*α* ^−/−^ macrophages, although slightly increased, were not significantly different compared to Inh*α* ^+/+^ (Figure 3(e)). Therefore, we can conclude that, in Inh*α* ^−/−^ mice, there is a significant reduction of MHCII expression only in cDCs but not in other hematopoietic stromal cells, such as pDCs or macrophages. These results confirm that these ligands do not always exert the same effects on different cell types.

### 3.3. Differentiation and Maturation of Thymic Epithelial Cells (TECs) Are Altered in Inh*α* ^**−**/**−**^ Mice

**Figure 4:**
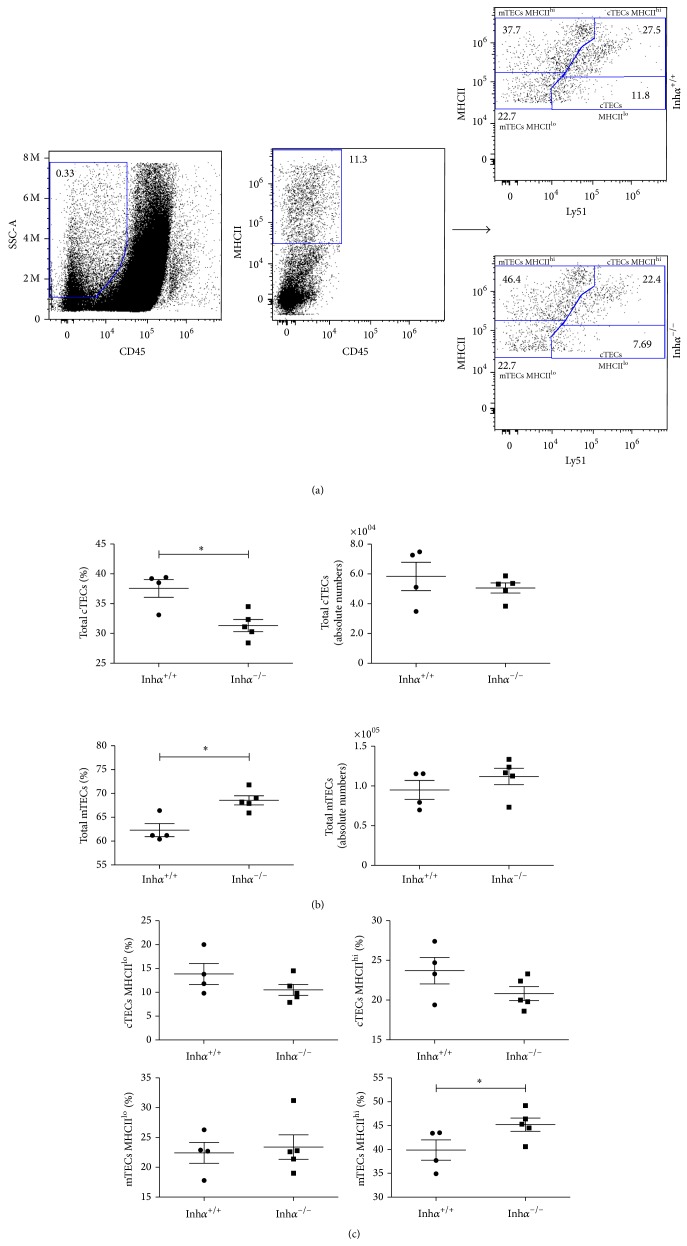
Inh*α* ^−/−^ mice show reduced numbers of thymic cTECs and express lower levels of MHCII. (a) The gating scheme to identify thymic epithelial cells is shown. From the CD45-gated subpopulation, Ly51 and MHCII markers are used to identify cortical (Ly51^+^ MHCII^+^) and medullary (Ly51^−^ MHCII^+^) epithelial cells. Among TEC subpopulations, both cortical and medullary, MHCII expression is used to distinguish immature (MHCII^lo^) and mature TECs (MHCII^hi^). Cortical and medullary epithelial cells subsets could be distinguished as Ly51^+^ MHCII^lo/hi^ and Ly51^−^ MHCII^lo/hi^, respectively (a). Dot blot that shows one representative experiment of a total of 9 (Inh*α* ^+/+^, *n* = 4; Inh*α* ^−/−^, *n* = 5) mice is shown. (b) The percentage and absolute numbers of total cTECs and mTECs are shown. A significant increase in the percentage of mTECs and a decrease in the percentage of cTECs in Inh*α* ^−/−^ compared to Inh*α* ^+/+^ mice. (c) A slight decrease in the percentage of cTEC MHC^lo^ (*P* = 0.09) and cTEC MHCII^hi^ (*P* = 0.07) and a significant increase in the percentage of mTEC^hi^ were detected in Inh*α* ^−/−^ compared to Inh*α* ^+/+^ mice. For this analysis, Inh*α* ^+/+^, *n* = 4 and Inh*α* ^−/−^, *n* = 5 were used. Statistical significance: ^∗^ *P* ≤ 0.05.

To determine whether MHCII expression was also affected in nonhematopoietic thymic cells, we next analyzed cTECs and mTECs from Inh*α* ^−/−^ and Inh*α* ^+/+^ mice, as previously described [27]. TEC comprises heterogeneous subpopulations of epithelial cells. Based on the staining with Ly51- and MHCII-antibodies, four major TEC subsets can be identified: Ly51^+^ MHCII^hi^ (cTEC MHCII^hi^), Ly51^+^ MHCII^lo^ (cTEC MHCII^lo^), Ly51^−^ MHCII^hi^ (mTEC MHCII^hi^), and Ly51^−^ MHCII^lo^ (mTEC MHCII^lo^) [47] (Figure 4(a)). Importantly, we found a significant decrease in the percentage of total cTECs (31.3% ± 1.02% versus 37.5% ± 1.5%, *P* = 0.009) and a concomitant increase in the percentage of mTECs in Inh*α* ^−/−^ mice (68.6% ± 0.9% versus 62.3% ± 1.4%, *P* = 0.006) (Figure 4(b)), while total cells numbers followed the same trend, although differences were not statistically significant (Figure 4(b)).

On the other hand, in Inh*α* ^−/−^ mice, the percentage of mTECs MHCII^hi^ were significantly increased in percentages (45.2% ± 1.4% versus 39.8% ± 2.1%, *P* = 0.03) (Figure 4(c)), and slightly increased in numbers (supplementary Figure  1A, Supplementary Material available online at http://dx.doi.org/10.1155/2015/837859) while cTECs (MHCII^lo^ and MHCII^hi^) tended towards a decrease both in percentage (Figure 4(c)) and numbers (suppl. Figure  1A). As cTECs and mTECs develop from a DEC205^+^ TEC bipotent common progenitor [14, 48], our results suggest that Inhibins may be negatively regulating mTEC versus cTEC differentiation, similarly to what has been recently reported for TGF-*β* [49]. Alternatively, the potential decrease in the percentage of cTEC MHCII^lo^ and concomitant increase in mTECs could be attributed to the presence of thymic epithelial cells precursors (TECP) within this Ly51^+^ MHCII^lo^ subpopulation (TEC^lo^), recently identified by Wong et al. [50], which are functionally relevant and may give rise both to mTECs and to cTECs MHCII^hi^ and which, in the absence of Inhibins, may be preferentially differentiating towards the mTEC lineage.

Interestingly, analysis of MHCII expression in the four TEC subpopulations demonstrated a significant reduction of MHCII expression in the cTEC MHCII^lo^ subpopulation from all Inh*α* ^−/−^ mice (suppl. Figure  1B), which correlated with a slight decrease in the percentage of the cTEC MHCII^lo^ (10.5% ± 1.1% versus 13.9% ± 2.2%, *P* = 0.09) and cTEC MHCII^hi^ (20.8% ± 0.9% versus 23.7% ± 1.7%, *P* = 0.07) subpopulations (Figure 4(c), left and right top panels, resp.). As it has been presumed that cTEC MHCII^lo^ may represent immature cells that later develop into cTEC MHCII^hi^ mature cTECs [51] we cannot exclude the possibility that cTEC maturation is also affected by the absence of Inhibins.

### 3.4. Inh*α* ^**−**/**−**^ Mice Show Increased Levels of Activin A

**Figure 5:**
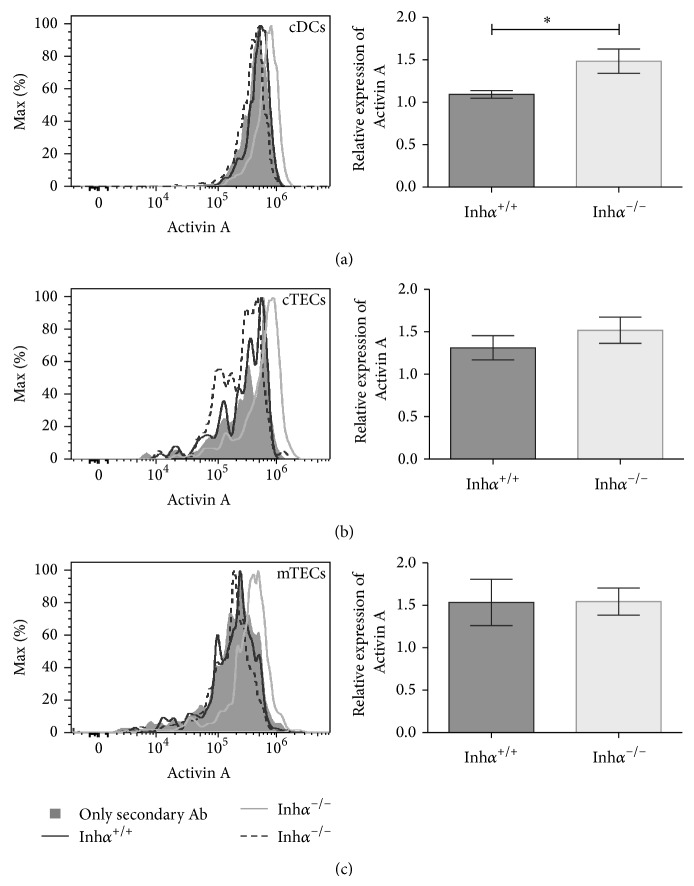
Inh*α* ^−/−^ thymic cDCs display increased intracellular levels of Activin A. The histograms show the intracellular Activin A expression in cDCs (a), cTECs (b), and mTECs (c) derived from Inh*α* ^+/+^ and Inh*α* ^−/−^ mice; for each histogram two Inh*α* ^−/−^ (gray and dashed lines) and one Inh*α* ^+/+^ mice (black line) were represented. (a, b, c right) summary of data showing the expression (MFI) of Activin A relative to the secondary antibody staining in the different cell subpopulation analyzed. One representative histogram from Inh*α* ^+/+^ (*n* = 5) and Inh*α* ^−/−^ (*n* = 6) mice analyzed is shown. Statistical significance: ^∗^ *P* ≤ 0.05.

Since Inhibins and Activins share the *β* subunit [52], targeted deletion of the Inhibin *α* subunit in mice not only removes Inhibins but leads to dysregulation of Activin expression, as a result of an increased *β*-*β* subunit assembly [3]. In this regard, it has been reported that female and male Inh*α* ^−/−^ mice present an overexpression of Activins A and B in serum as early as 7 weeks of age, with the gonadal sex cord-stromal tumors being recognized as their main source of these ligands [3, 53]. In addition, previous reports demonstrated that DCs can produce Activins that act in an autocrine manner down modulating DC maturation (reviewed in [5]). To investigate the potential involvement of Activins in MHCII and CD86 downregulation, we analyzed the production of Activin A in cDCs (Figure 5(a)), cTECs (Figure 5(b)), and mTECs (Figure 5(c)) from Inh*α* ^−/−^ and Inh*α* ^+/+^ mice by flow cytometry. As shown in Figure 5 and summarized in Table 1, although all Inh*α* ^−/−^ mice presented decreased levels of MHCII in cDCs, only 50–67% of them showed detectable intracellular Activin A in either cDCs or TECs. When detected, Activin A expression was significantly higher in cDCs from Inh*α* ^−/−^ compared to Inh*α* ^+/+^ mice (*P* = 0.03) and slightly increased in other stromal subpopulations. These results indicate that there is no correlation between intracellular Activin levels and MHCII/CD86 downregulation, suggesting that autocrine production of Activins by DCs is not responsible for the effects observed in Inh*α* ^−/−^ stromal cells. However, we cannot rule out the possibility that Activins secreted by other stromal cells (such as fibroblasts or endothelial cells) may be acting in a paracrine manner affecting the maturation of DCs.

As Inh*α* ^−/−^ mice are known to overexpress Activins in the serum, we decided to evaluate the seric levels of Activin A in our 2-week-old Inh*α* ^−/−^ mice using a high-sensitivity ELISA kit. We found that the Activin A levels in Inh*α* ^−/−^ mice were elevated 1.5-fold compared with Inh*α* ^+/+^ mice (1664 ± 86.41 pg/mL versus 1117 ± 39.52 pg/mL, *P* = 0.0005) (suppl. Figure  2) however, this increment was much lower than the one previously reported in Inh*α* ^−/−^ female mice of 10 to 20 weeks of age (~20-fold increase), with well-developed gonadal tumors [3]. Moreover, in two different transgenic models of Activin A overexpression, the biological effects observed* in vivo* were also associated with an increment in the levels of Activin similar to that reported by Matzuk et al. (20 to >100 fold) [54, 55].

### 3.5. CD4 SP Selection Is Impaired and nTreg Development Increased in Inh*α* ^**−**/**−**^ Mice

DP thymocytes bearing TCRs able to recognize peptide-MHCII complexes with low avidity are positively selected and differentiate into CD4SP T cells [13]. Additionally, high-affinity MHCII-peptide-TCR binding induces thymocyte clonal deletion (negative selection) and the generation of nTregs [56]. Indeed, it is now well accepted that the TCR signalling threshold has a key role in the cell fate of the thymocyte [57].

**Figure 6:**
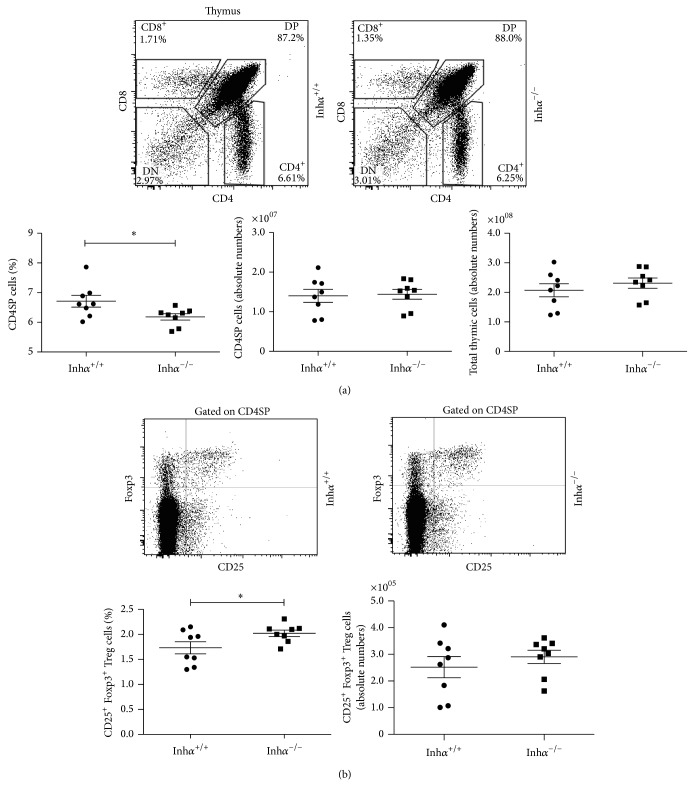
Decreased percentage of CD4SP and increased levels of Tregs in the thymus of Inh*α* ^−/−^ mice. Thymocytes from 2-week-old Inh*α* ^+/+^ and Inh*α* ^−/−^ mice were isolated, counted, and stained to CD4, CD8, CD25, and Foxp3. (a) Dot plots show the percentage of thymocyte subpopulations. Graphs represent the absolute cell numbers of thymocytes and the percentage and absolute cell numbers of CD4SP. (b) Dot plots show the percentage of CD25^+^ Foxp3^+^ cells gated in CD4SP and the graphs depict the percentage of CD25^+^ Foxp3^+^ cells and the absolute numbers of this subpopulation among CD4^+^ cells. A representative experiment is shown. Mean values ± SEM are shown (*n* = 8 per group). Statistical significance: ^∗^ *P* ≤ 0.05.

As MHCII expression is diminished in Inh*α* ^−/−^ cTECs, one may predict that an altered T cell repertoire would be positively selected, possibly resulting in decreased CD4^+^ T cell selection. Consequently, the following was to compare the numbers and percentages of the DN, DP, CD4SP, and CD8SP thymocyte subpopulations between Inh*α* ^−/−^ and Inh*α* ^+/+^ mice (Figure 6(a) and suppl. Figure  3). As predicted, the analysis showed a significant reduction in the percentage of CD4SP cells in Inh*α* ^−/−^ compared with Inh*α* ^+/+^ mice (6.2% ± 0.1% versus 6.7% ± 0.2%, *P* = 0.02) (Figure 6(a)). The absolute cell numbers of total thymocytes and CD4SP cells showed no significant differences (Figure 6(a) bottom panels). Additionally, we observed that the decrease in CD4SP in Inh*α* ^−/−^ mice was accompanied by a slight increase (although not significant) in the percentage and absolute cell numbers of DP thymocytes while no changes were observed in the percentages and cell numbers of CD8SP or DN subpopulations of Inh*α* ^−/−^ compared with Inh*α* ^+/+^ mice, respectively (suppl. Figure  3). Analysis of thymocyte subpopulations had previously been performed in Inh*α* ^−/−^ fetal thymic organ cultures, showing no statistical differences in the percentages and cell numbers of all thymocyte subpopulations, including the CD4SP [25]. These dissimilar results can be explained by the use of different experimental systems. Thus,* in vitro* differentiation of ED14-FTOC does not completely achieve the same proportions of thymocyte subpopulations as those observed in thymi from 2-week-old (suppl. Figure  3) and adult mice [58].

Our results are consistent with previous studies that show the pivotal role of MHCII expressed on cTECs in the positive selection of CD4^+^ T lymphocytes. Namely, Waldburger et al. [59] through knocking out the pIV promoter of* Mhc2ta* gene (encoding CIITA), selectively abrogated the expression of MHCII in cTECs resulting in a 7–10-fold reduction in the percentage of CD4SP in the thymus [59]. Additionally, Cathepsin-L deficient mice, a cTEC-specific lysosomal protease that regulates degradation of the invariant chain [39], exhibited changes in the MHCII-peptide repertoire leading to inefficient selection of CD4SP thymocytes [60, 61].

Among CD4SPs, nTregs (CD25^+^ Foxp3^+^) are known to be selected under conditions of high avidity. As shown in Figure 6(b), Inh*α* ^−/−^ mice showed a significant increase in the percentage of nTregs compared to Inh*α* ^+/+^ mice (2.02% ± 0.06% versus 1.73% ± 0.12%, *P* = 0.03), although the absolute cell numbers of this subpopulation were not significantly affected. This indicated that the nTreg/CD4SP ratio is altered in Inh*α* ^−/−^ mice, favoring the selection of nTregs. Therefore, the reduction in high avidity interactions presumably occurring in Inh*α* ^−/−^ mice appears to lower the threshold of selection, shifting the balance from negative selection to nTreg development, in agreement with the avidity model of selection [62].

In an attempt to dissect the role of the avidity in negative selection versus nTreg differentiation, Hinterberger et al. generated a mouse model in which antigen presentation was selectively attenuated in mTECs through RNA interference-mediated knockdown of MHCII on Aire-expressing cells. The results showed a decreased negative selection and increased generation of specific nTreg cells [63]. Although this study enhances only the role of mTECs in the induction of nTregs in TCR-peptide-MHCII low avidity interactions, this could also be the case for thymic DCs. In support of the above, experiments carried out* in vivo* by Atibalentja et al., through systemic administration of varying concentrations of hen egg-white lysozyme (HEL), which was rapidly processed and presented in the thymus solely by DCs, demonstrated that although low concentrations of HEL were able to induce both negative selection of specific TCR transgenic conventional T cells and antigen specific nTreg development, the greatest increase in nTreg absolute numbers occurred at doses below that required for complete negative selection [64].

In summary, our data demonstrate that Inhibins regulate stromal cell differentiation favoring the development of cTECs versus mTECs and suggest a potential role for Inhibins in the homing of pDCs to the thymus. In addition, the absence of Inhibins impairs the maturation of cTECs and cDCs, affecting the avidity of TCR-peptide-MHCII interactions, and thereby impacts the T cell selection process leading to impaired positive selection of CD4SP and increased nTreg development.

## Supplementary Material

Supplementary Figure 1. (A) The absolute cell numbers of TEC subpopulations from Inhα^−/−^ and Inα^+/+^ mice showed a slight decrease in the numbers of cTECs and slight increase in mTECs, between Inhα^−/−^ and Inhα^+/+^ mice. (B) MHCII expression showed a significant reduction within the cTEC MHCII^lo^ subpopulation from Inhα^−/−^ mice compared to Inhα^+/+^ mice.
Supplementary Figure 2. The measurement of serum Activin A levels by ELISA showed increased levels of Activin A in all Inhα^−/−^ mice compared to Inhα^+/+^ mice.
Supplementary Figure 3. The percentage of CD4SP thymocytes, but not the absolute cell numbers, show a significant reduction in Inhα^−/−^ mice compared to Inhα^+/+^mice.

## Figures and Tables

**Table 1:** Relative intracellular expression of Activin A in thymic cDCs and thymic epithelial cell subpopulations from Inh*α* ^+/+^ and Inh*α* ^−/−^ mice.

Stromal cell	Genotype		Activin	
		D	ND	Relative intracellular expression ± SEM
cDC	Inh*α* ^+/+^	4	1	1.093 ± 0.0445
	Inh*α* ^−/−^	3	3	1.484 ± 0.1429^*^

Total cTECs	Inh*α* ^+/+^	3	2	1.311 ± 0.1426
	Inh*α* ^−/−^	4	2	1.518 ± 0.1545

cTECs MHCII^hi^	Inh*α* ^+/+^	3	2	1.485 ± 0.2758
	Inh*α* ^−/−^	4	2	1.650 ± 0.2196

cTECs MHCII^lo^	Inh*α* ^+/+^	2	3	1.235 ± 0.0212
	Inh*α* ^−/−^	3	3	1.400 ± 0.1623

Total mTECs	Inh*α* ^+/+^	3	2	1.534 ± 0.2736
	Inh*α* ^−/−^	4	2	1.544 ± 0.1593

mTECs MHCII^hi^	Inh*α* ^+/+^	4	1	1.491 ± 0.2969
	Inh*α* ^−/−^	4	2	1.649 ± 0.3075

mTECs MHCII^lo^	Inh*α* ^+/+^	3	2	1.379 ± 0.1593
	Inh*α* ^−/−^	4	2	1.452 ± 0.1330

Intracellular levels of Activin were measured by flow cytometry, using surface markers to differentiate different stromal cell subpopulations: cDCs (CD45^+^ CD11c^+^ MHCII^+^); cTECs (CD45^−^ MHCII^+^ Ly51^+^); mTECs (CD45^−^ MHCII^+^ Ly51^−^). MHCII high and low subpopulations were also analyzed to define mature versus immature subpopulations, respectively. Relative intracellular expression of Activin A was calculated normalizing the MFI from each sample to the MFI of the secondary antibody control from mice in column D. D: number of mice with detectable Activin levels; ND: not detectable. Statistical significance: ^∗^ *P* ≤ 0.05.
