# Supplementary material for: Inhibins Tune the Thymocyte Selection Process by Regulating Thymic Stromal Cell Differentiation
Source: J Immunol Res. 2015 Apr 20;2015:837859. doi: 10.1155/2015/837859 (PMC4418002; doi:10.1155/2015/837859)
Supplement: Supplementary file 1 — Supplementary Figure 1. (A) The absolute cell numbers of TEC subpopulations from Inhα−/− and Inα+/+ mice showed a slight decrease in the numbers of cTECs and slight increase in mTECs, between Inhα−/− and Inhα+/+ mice. (B) MHCII expression showed a significant reduction within the cTEC MHCIIlo subpopulation from Inhα−/− mice compared to Inhα+/+ mice. Supplementary Figure 2. The measurement of serum Activin A levels by ELISA showed increased levels of Activin A in all Inhα−/− mice compared to Inhα+/+ mice. Supplementary Figure 3. The percentage of CD4SP thymocytes, but not the absolute cell numbers, show a significant reduction in Inhα−/− mice compared to Inhα+/+mice. [file 837859.f1.pdf]

Supplementary Figure 1.

(A)

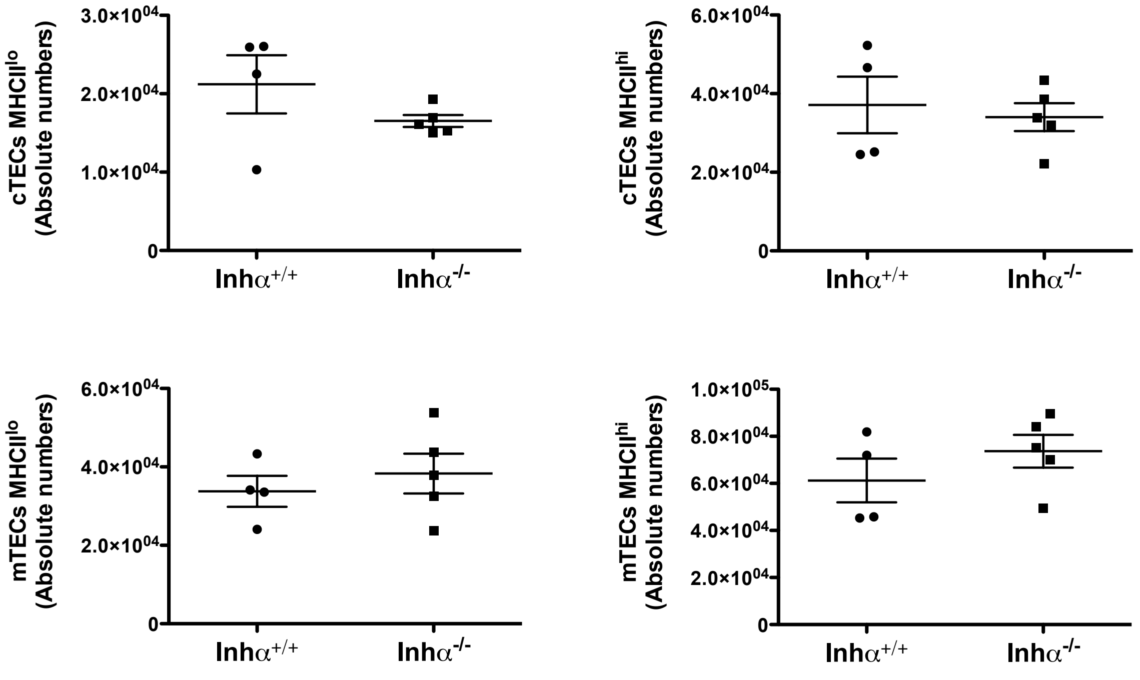

(B)

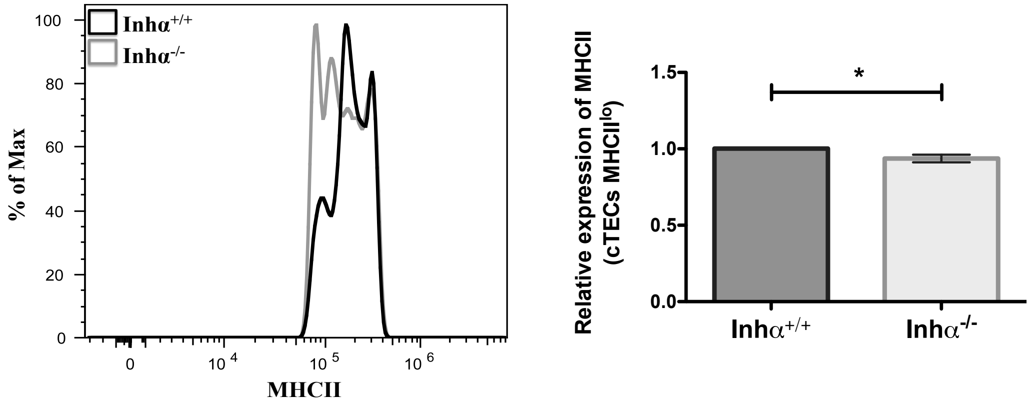

(A) The absolute cell numbers of TEC subpopulations from Inh$\alpha^{-/-}$ (n=5) and In$\alpha^{+/+}$ (n=4) mice are shown. A slight decrease in cTECs and slight increase in mTECs (although not significant) was observed when comparing cell numbers between Inh$\alpha^{-/-}$ and Inh$\alpha^{+/+}$ mice. (B) MHCII expression present in cTECs from Inh$\alpha^{+/+}$ or Inh$\alpha^{-/-}$ mice was calculated and reported as relative MFI, showing a significant reduction within the cTEC MHCII^{lo} subpopulation from Inh$\alpha^{-/-}$ mice. Statistical significance: *$p \leq 0.05$.

Supplementary Figure 2.

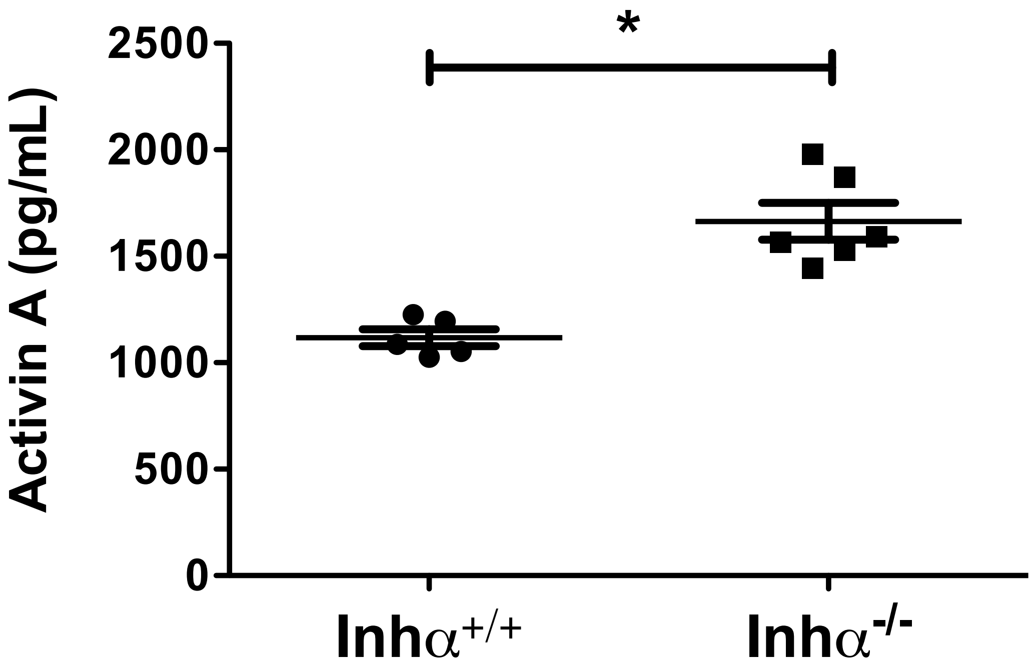

Activin A serum levels, measured by ELISA from both Inhα$^{+/+}$ and Inhα$^{-/-}$ mice. As shown, increased levels of Activin A were present in all Inhα$^{-/-}$ mice compared to Inhα$^{+/+}$ mice (1664 ± 86.41 pg/mL, n=6 versus 1117 ± 39.52 pg/mL, n=5, p=0.0005). Statistical significance: *$p \leq 0.05$.

Supplementary Figure 3.

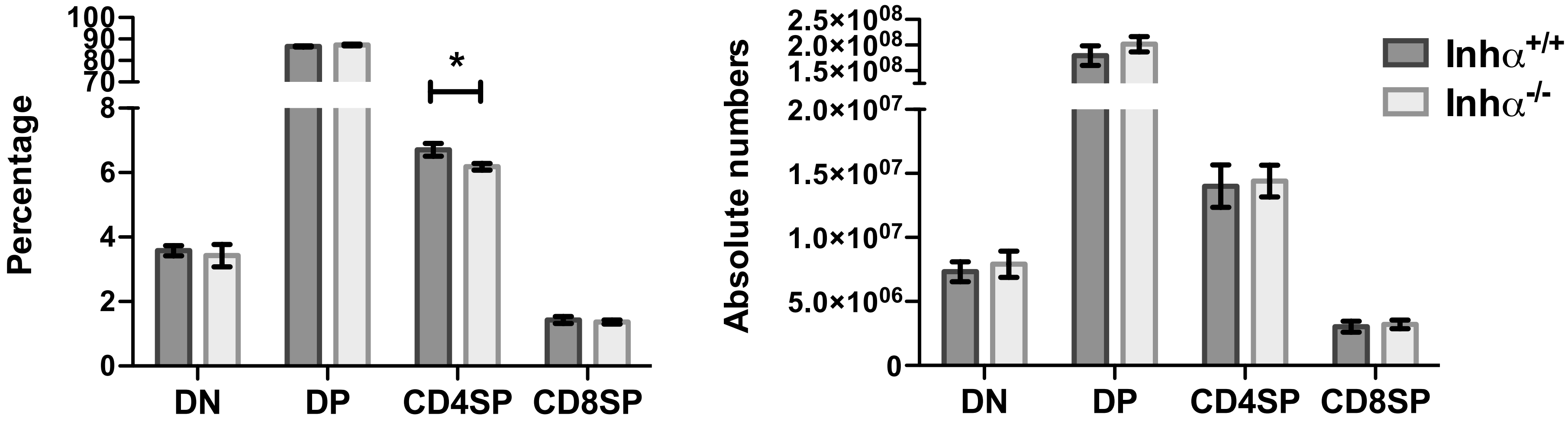

The percentage and absolute numbers of thymocyte subpopulations from Inhα$^{+/+}$ and Inhα$^{-/-}$ mice are shown. For all subpopulations total numbers of thymocytes were not significantly different between Inhα$^{+/+}$ and Inhα$^{-/-}$ mice (with a slight increase in DPs). A significant decrease in the percentage of CD4SP thymocytes from Inhα$^{-/-}$ mice (6.2% ± 0.1%, *n*=8, *p*=0.02) was observed compared to Inhα$^{+/+}$ mice (6.7% ± 0.2%, *n*=8). Statistical significance: **p*≤0.05.
